# Study protocol: population screening for colorectal cancer by colonoscopy or CT colonography: a randomized controlled trial

**DOI:** 10.1186/1471-230X-10-47

**Published:** 2010-05-19

**Authors:** Thomas R de Wijkerslooth, Margriet C de Haan, Esther M Stoop, Marije Deutekom, Paul Fockens, Patrick MM Bossuyt, Maarten Thomeer, Marjolein van Ballegooijen, Marie-Louise Essink-Bot, Monique E van Leerdam, Ernst J Kuipers, Evelien Dekker, Jaap Stoker

**Affiliations:** 1 Department of Gastroenterology and Hepatology, Academic Medical Centre, Amsterdam, the Netherlands; 2 Department of Radiology, Academic Medical Centre, Amsterdam, the Netherlands; 3 Department of Gastroenterology and Hepatology, Erasmus MC University Medical Centre, Rotterdam, the Netherlands; 4 Department of Social Medicine, Academic Medical Centre, Amsterdam, the Netherlands; 5 Clinical Epidemiology, Biostatistics and Bioinformatics, Academic Medical Centre, Amsterdam, the Netherlands; 6 Department of Radiology, Erasmus MC University Medical Centre, Rotterdam, the Netherlands; 7 Department of Public Health, Erasmus MC University Medical Centre, Rotterdam, the Netherlands; 8 Department of Internal Medicine, Erasmus MC University Medical Centre, Rotterdam, the Netherlands

## Abstract

**Background:**

Colorectal cancer (CRC) is the second most prevalent type of cancer in Europe. Early detection and removal of CRC or its precursor lesions by population screening can reduce mortality. Colonoscopy and computed tomography colonography (CT colonography) are highly accurate exams and screening options that examine the entire colon. The success of screening depends on the participation rate. We designed a randomized trial to compare the uptake, yield and costs of direct colonoscopy population screening, using either a telephone consultation or a consultation at the outpatient clinic, versus CT colonography first, with colonoscopy in CT colonography positives.

**Methods and design:**

7,500 persons between 50 and 75 years will be randomly selected from the electronic database of the municipal administration registration and will receive an invitation to participate in either CT colonography (2,500 persons) or colonoscopy (5,000 persons) screening. Those invited for colonoscopy screening will be randomized to a prior consultation either by telephone or a visit at the outpatient clinic. All CT colonography invitees will have a prior consultation by telephone. Invitees are instructed to consult their general practitioner and not to participate in screening if they have symptoms suggestive for CRC. After providing informed consent, participants will be scheduled for the screening procedure. The primary outcome measure of this study is the participation rate. Secondary outcomes are the diagnostic yield, the expected and perceived burden of the screening test, level of informed choice and cost-effectiveness of both screening methods.

**Discussion:**

This study will provide further evidence to enable decision making in population screening for colorectal cancer.

**Trial registration:**

Dutch trial register: NTR1829

## Background

Colorectal cancer (CRC) is the second most prevalent type of cancer in Europe. In 2006, 412,900 persons were diagnosed with CRC and 207,400 persons died from the disease[1]. In the Netherlands, more than 4,700 persons died as a result of CRC in 2006[2,3]. The prognosis of patients with CRC depends on the clinical and pathological stage at the time of diagnosis. Early detection of CRC reduces CRC-related mortality; early detection and removal of its precursor lesions -adenomas- reduces both the incidence and mortality of CRC[4]. Thus, population screening of asymptomatic average risk persons can reduce the mortality rate [5-8]. In addition, given the high, rapidly rising costs of treatment of CRC, screening has actually become cost saving[9].

The currently available options for colorectal cancer screening are stool based tests (guaiac and immunochemical faecal occult blood tests and faecal DNA tests) and structural exams (flexible sigmoidoscopy, colonoscopy, double contrast barium enema and CT colonography). Colon capsule is another technique that examines the entire colon. However, this modality is currently not accepted for CRC screening[10,11].

Randomized clinical trials evaluated guaiac-based FOBT (gFOBT) screening during a 10-year screening period and showed reduction in CRC-mortality[12]. Immunochemical FOBT (iFOBT) is considered a superior screening test, because of its better reproducibility and acceptance. Detection rates for advanced adenomas and cancer are higher compared to gFOBT[13]. Furthermore, iFOBT screening offers the option to select a cut-off level matching the optimal performance of the test in a given population with the available colonoscopy capacity[14,15]. A new method of CRC screening that tests DNA markers in stool (sDNA) may be a promising tool for screening in the future. At this moment sDNA does not provide any advantages as a screening method compared with FOBT. Flexible sigmoidoscopy (FS) is an endoscopic procedure, in which the distal 40-60 cm of the colon is inspected. Total colonoscopy is advised in case of positive findings. FS has a threefold higher detection rate for advanced neoplasia compared with FOBT[16,17]. Double contrast barium enema (DCBE) and capsule endoscopy can be considered as inferior screening modalities than colonoscopy. Accuracy was considerably lower and these techniques were not tested in an average risk screening population[18,19].

Colonoscopy and CT colonography are structural exams allowing inspection of the entire colon. Colonoscopy is widely accepted as the clinical reference standard for the detection of colorectal neoplasia and has the advantage that removal of adenomas or early cancer can be performed during the same procedure. All other screening procedures require colonoscopy for confirmation of a diagnosis and, if applicable, therapy (polypectomy) in case of a positive test result. Furthermore, colonoscopy screening can be performed with long intervals since the risk of developing CRC after a negative colonoscopy remains decreased for more than 10 years[20,21]. Despite the excellent sensitivity and specificity of colonoscopy, miss rates of 2.1% for large adenomas (≥ 10 mm) are reported in tandem colonoscopy studies[22]. A disadvantage of colonoscopy as a screening method is its complication rate of 0.1 to 0.3% including post polypectomy bleeding and perforation [23-25]. Secondly, colonoscopy is a burdensome procedure for which full bowel cleansing is necessary. These factors together explain the limited uptake of colonoscopy as a primary screening test and the insufficient use of colonoscopy for surveillance after previous adenoma removal[26]. Finally, colonoscopy capacity is a limiting factor for its widespread use as primary screening test.

CT colonography is a less invasive full colonic exam which can be performed with limited bowel preparation and could therefore be a good alternative in a screening setting [27-29]. CT colonography has been demonstrated to have a high sensitivity for the detection of CRC (96%)[30]. A large screening trial evaluating CT colonography and same day colonoscopy studied 1233 asymptomatic individuals and reported high sensitivity (94%) and specificity (96%) per patient for large adenomas (≥10 mm). Sensitivity and specificity for adenomas larger than or equal to 6 mm was 89% and 80% respectively[31]. The diagnostic yield for advanced neoplasia of CT colonography (3.2%) was comparable to that of colonoscopy (3.4%)[32]. The risk of complications is extremely low, there were no perforations or other serious complications in a large CT colonography screening cohort[32]. CT colonography with limited bowel preparation has a lower burden and is preferred by patients compared to regular CT colonography[33]. A disadvantage of CT colonography is the exposure of individuals to ionizing radiation. However, the chances of radiation induced malignancy are considered very low, especially when a low dose protocol is used. The detection of extracolonic findings in CT colonography could be beneficial, but risks and costs associated with false positives and inconsequential findings may be substantial. Of all available screening tests for CRC, colonoscopy and CT colonography are the most accurate exams.

The optimal screening test for CRC is still a subject of fierce debate. FOBT and FS are suboptimal tests whereas full colonic exams, such as colonoscopy and CT colonography are associated with risks, costs and high workload. An evidence-based estimate of the participation rate is essential for making predictions of the effectiveness and costs of a population screening programme.

In the Netherlands, population based screening trials by gFOBT, iFOBT and FS have already been performed. Studies that investigated stool-based tests as a screening method reported participation rates of 47-50% (gFOBT) and 60-62% (iFOBT)[13,17]. FS-screening had a lower participation rate of 32%[17].

We will conduct a study evaluating the participation rate of both colonoscopy and CT colonography as a screening method in a population-based programme. Since colonoscopy is a more invasive procedure, participation in a colonoscopy screening programme can be expected to be lower than in FOBT and FS screening[34]. On the other hand, FOBT and FS screening should be repeated every 2 and 5 years and participation over a 10 years period could be considerably lower than for a single round of screening[6]. Participation rates might be influenced by the burden of the screening procedure itself and its bowel preparation. We will therefore evaluate the expected and actual perceived burden of both screening methods, as well as the reasons to participate or not in the screening programme.

In most settings, all patients planned for colonoscopy are invited for a prior consultation at the outpatient clinic. In an effort to reduce the number of patients not attending for colonoscopy, *Rodger J et al. *introduced a consultation by telephone in a FOBT positive population in a CRC screening programme and found a significant reduction of 14.1%[35]. Therefore, we hypothesize that a consultation by telephone instead of an appointment at the outpatient clinic prior to colonoscopy could contribute to a higher participation rate in primary colonoscopy screening. Screening invitees should be enabled to make a well-informed decision to participate or not and we will evaluate the level of informed choice in the decision making process[36,37]. Since the expected costs and workload of a population based screening programme by colonoscopy and CT colonography are serious concerns, we will evaluate the cost-effectiveness and feasibility and compare these with the other available screening techniques in the Netherlands.

## Methods and design

### Objectives

#### Primary objective

To compare the participation rates in a population-based screening programme for colorectal cancer by primary colonoscopy and CT colonography.

#### Secondary objectives

• To compare the diagnostic yield of both screening techniques (detection rates of cancer, advanced adenomas and adenomas).

• To compare the expected and perceived burden of colonoscopy and CT colonography.

• To compare the cost-effectiveness and feasibility of colonoscopy and CT colonography as screening methods.

• To compare the level of informed choice in the decision-making process for (non-) participation in colonoscopy and CT colonography screening.

### Study design

**Figure 1:**
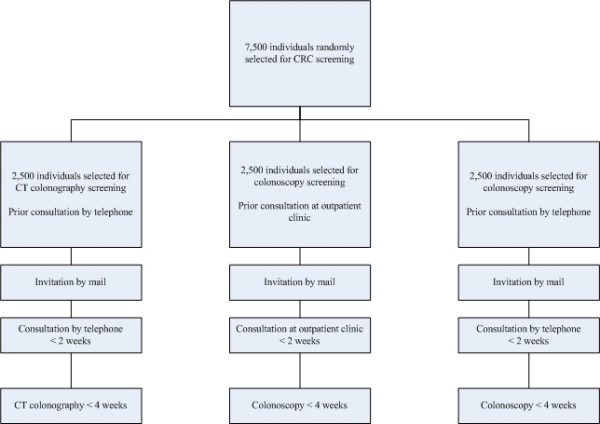
**Design of the screening trial**. Flow chart.

This study will be a two-centre randomized controlled trial. A cohort of 7,500 persons of the Amsterdam and Rijnmond region will be randomly selected from the electronic database of the regional municipal administration registrations (Gemeentelijke Basis Administratie (GBA)). In total, 2,500 persons will be randomly selected to receive an invitation for colonoscopy screening with a prior consultation by telephone. Another 2,500 persons will be randomly selected to receive an invitation for colonoscopy screening with a prior consultation at the outpatient clinic. A third group of 2,500 persons will be randomly selected to receive an invitation for CT colonography, with a prior consultation by telephone [figure 1]. Randomization will be performed per household, stratified for age, sex and socio-economic status (SES).

### Study population

Our study population consists of 7,500 individuals between 50 and 75 years of age living in our target areas. The selected areas comprise all SES-categories (very low, low, average, high and very high) and are based on data of Statistics Netherlands[3]. The target areas will not have been selected for previous pilot trials on CRC screening. Individuals with CRC symptoms in the previous three months (rectal blood loss and/or changed bowel habits) are advised in our information leaflet to consult their general practitioner and to not participate in screening. Persons who underwent full colonic examination in the previous 5 years (complete colonoscopy and/or double contrast barium enema) are also instructed not to participate and are excluded from the screening programme, as well as individuals planned for surveillance colonoscopy, because of personal history of colorectal cancer, colonic adenomas or inflammatory bowel disease (IBD)). Persons with a severe or terminal disease with a life-expectancy of less than 5 years are excluded. Although pregnancy is not likely in this cohort, pregnant women are also excluded. For CT colonography, individuals exposed to ionizing radiation for research purposes within the previous 12 months and individuals with hyperthyroidism are excluded.

### Invitation procedure

Similar to our previous CRC screening trials, a specialized database will be used for the logistics of the invitational procedure. All invitations will be sent out between June 2009 and September 2010 by the regional Comprehensive Cancer Centres in Amsterdam and Rotterdam.

Members of the target population will receive a pre-announcement, followed by an invitation for the screening programme two weeks later, both by mail. Information leaflet and reply card are sent together with the invitation. All invitees have three options to respond: by returning the reply-card, by calling the Comprehensive Cancer Centre or by sending an e-mail. The Comprehensive Cancer Centre will make an appointment for a prior consultation. All non-respondents will receive a reminder by mail 4 weeks after the initial invitation.

### Information leaflet

Three different information leaflets will be used, one for each arm. The information leaflet is an updated version of the leaflet used in previous CRC screening trials in the Amsterdam and Rijnmond region and modified for the screening methods studied. The leaflet is based on the principles of informed-choice, aiming to enable all invited persons to make a well-informed decision whether or not to participate. The leaflet consists of information on CRC in general, the advantages and possible risks of the screening method and on the follow-up in case of a positive test result.

### Prior consultation

Persons allocated to colonoscopy screening are invited for a prior consultation either by telephone or at the outpatient clinic. Prior to CT colonography, all invited individuals will receive a consultation by telephone. During the consultation, they are informed about the screening procedure, bowel preparation, possible risks and the follow-up in case of a positive test result. A standardized questionnaire is used to check on contra-indications and/or exclusion criteria. If additional information is needed regarding a possible exclusion criterion or contra-indication for the screening procedure, the general practitioner or medical specialist will be contacted for further information or the individual selected for intake by telephone will be invited for a visit at the outpatient clinic. Individuals without contraindications will be scheduled for screening at the regional CRC-screening centre. Subsequently, all colonoscopy screening participants will be asked to perform an immunochemical FOBT.

### Informed consent

Informed consent will be discussed during the prior consultation. All participants are instructed to return the informed consent form by mail before the scheduled screening procedure.

### Colonoscopy

All colonoscopies will be performed at one of the two participating centres by gastroenterologists with an experience of 500 or more colonoscopies. The colonoscopy will be performed taking the standard quality aspects into account defined by the American Society for Gastrointestinal Endoscopy[38]. All participants are prepared by a low fibre diet and by oral intake of 2 L of transparent fluid and 2 L of hypertonic polyethylene glycol solution (Moviprep; Norgine bv, Amsterdam, The Netherlands) at home. The procedures are performed under conscious sedation using intravenous midazolam (Dormicum, Actavis, Baarn, The Netherlands) and fentanyl (Bipharma, Weesp, The Netherlands) at the discretion of the participant and the endoscopist. In case of poor bowel preparation the colonoscopy is interrupted and postponed. Cecal intubation by the colonoscope is confirmed by still images of the appendiceal orifice and ileocecal valve or by intubation of the ileum. Insertion and withdrawal times will be separately recorded. At the start of withdrawal of the endoscope, butylscopalamine bromide (Buscopan, Boehringer Ingelheim bv, Alkmaar, The Netherlands) will be given intravenously at the discretion of the endoscopist to reduce colonic motility and repeated if necessary. Inspection on withdrawal of the endoscope will be at least 6 minutes[39]. All detected lesions will be removed during the same procedure if possible. If immediate endoscopic treatment is impossible, biopsies will be obtained and pathological assessment of these tissue samples will provide a definitive diagnosis.

#### Lesions

Of all detected lesions during colonoscopy the size (millimetres), morphology (sessile, pedunculated, flat or depressed), localization (distance from the anus, segment of the colon) and macroscopic aspect (hyperplastic, adenomatous, carcinomatous) will be noted. The size of each lesion is measured using an open biopsy forceps with 7 mm span. Furthermore, data on diagnostic or therapeutic procedure (biopsy, piecemeal polypectomy, coagulation or cold-snare total polypectomy), macroscopic involvement of margins, use of saline and/or epinephrine, and time needed for polypectomy will be recorded.

#### Pathology

Histology will be defined according to the Vienna criteria[40]. Dysplasia will be defined as either low grade or high grade and all lesions will be classified into hyperplastic, serrated, tubular, tubulovillous, villous or carcinoma lesion. Histopathology will be processed and stained using standard methods and will be evaluated by two expert pathologists (one in each centre). All advanced neoplasias and a random selection of 10% of all other lesions evaluated in each centre will be revised by the pathologist of the other centre. In case of inconsistency, the slides will be reviewed together to come to a definitive diagnosis.

### CT colonography

The preparation for CT colonography includes two times 50 mL of iodinated contrast agent (Telebrix, Guerbet, Aulnay sous Bois, France) for tagging on the day prior to the study and 50 mL 1.5 hour before the examination, combined with a low-residue diet. This regimen resulted in high image quality not distinct from a full bowel preparation[41]. Bowel preparation with tagging is now indicated as best practice in the recently published international CT colonography standards[42]. Colonic distension will be obtained with an automatic carbon dioxide insufflator (PROTOCO2L, Bracco, EZEM, Lake Success, USA) after intravenous administration of 1 ml butylscopalamine bromide. If butylscopalamine is contraindicated, 1 mg of glucagonhydrochloride (GlucaGen, Novo Nordisk A'S, Bagsvaerd, Denmark) will be used intravenously. If both butylscopalamine bromide and glucagonhydrochloride are contraindicated, CT colonography examination will be performed without bowel relaxants. CT colonography will be performed with a 64-slice CT scanner in one of the two participating centres by qualified and experienced personnel. Images will be obtained in both the supine and prone position, using a low dose protocol with the following specifications: collimation 64 × 0.625 mm, slice thickness 0.9 mm, reconstruction interval 0.7 mm, tube voltage 120 kV and 25 ref mAs supine and prone.

Each CT colonography is evaluated by one of the three experienced physicians (two abdominal radiologists, one research fellow) as well as two of the four experienced technicians (prior experience ≥200 CT colonography examinations with colonoscopic verification). Image processing and interpretation is performed with the use of a non-commercially available CT colonography workstation (View Forum, Demo version R6.1V1L1, Philips, Eindhoven, The Netherlands). Primary 2D read will be used, with endoluminal 3D problem solving and the use of computer assisted detection (CAD) as a secondary read. Lesion size will be measured on 3D images, unless there is too much faecal material around the polyp, in that case 2D measurements will be used.

#### Lesions

Both intra- and extracolonic findings will be recorded. A true positive intracolonic CT colonography finding is defined as a CT colonography lesion of at least 6 mm that is found in the same or adjacent segment on the colonoscopy with the size of the lesion at least <50% margin of error.

Diminutive polyps (5 mm and smaller) will be ignored as the chance for malignancy is very low. Of all intracolonic lesions ≥ 6 mm, data on certainty (25%, 50%, 75% and 100%), size, location and morphology (sessile, pedunculated, flat) will be documented. If referred for colonoscopy, CT colonography results will be verified using segmental unblinding and histopathology.

Extracolonic structures will be examined using the C-RADS classification[43]. An extracolonic lesion of C-RADS E3 (likely unimportant finding, incompletely characterized) or E4 (potentially important finding) is considered to be clinically relevant.

### Complications

Complications will be registered until 30 days after the screening procedure. Complications of CT colonography are defined as all complications occurring during CT colonography as well as all complications of colonoscopy in CT colonography positives. Of all complications the timing, severity, relation to the procedure, treatment and outcome will be reported. All participants will be interviewed by telephone to ensure accurate complication registry.

### Follow-up after positive test result

#### Colonoscopy

All participants will be informed about the result of colonoscopy on the day of the procedure. In the case of polyps or cancer, histopathological assessment of tissue samples will provide a definitive diagnosis and participants will be informed about the results within 2 weeks. Advice regarding surveillance colonoscopy will be given to persons according to the Dutch Institute for Healthcare Improvement (CBO) consensus[44]. In case of cancer, the patient is invited at the outpatient clinic and referred to a gastroenterologist or surgeon for further treatment. The general practitioner and participant will receive a letter about the results of the colonoscopy and corresponding follow-up if needed after two weeks.

#### CT colonography

All participants will be informed by telephone about the results of CT colonography within 2 weeks. Both the participant and the general practitioner will receive a letter by mail with the findings at CT colonography. Individuals with one or more lesions ≥10 mm at CT colonography will be referred for colonoscopy, while individuals with only lesions 6-9 mm in size will be advised to undergo surveillance CT colonography. Surveillance CT colonography will be advised after 1.5 years when there are three or more 6-9 mm lesions and 3 years when there are one or two 6-9 mm lesions. If referral for colonoscopy is needed, the participant will be invited for a visit at the outpatient clinic where information is given on the consequences of the positive test result and if there are no contraindications a colonoscopy will be advised. If the participant consents, a colonoscopy will be performed within the following two weeks. Patients with relevant extracolonic findings will be invited at the outpatient clinic and referred for corresponding follow-up[43].

### Questionnaires

At different time-points, questionnaires will be provided. The first questionnaire is sent after the prior consultation to all subjects scheduled for the screening test to assess the following items: demographic and socioeconomic status, satisfaction of the prior consultation, reasons for participation, level of informed choice and expected burden of the screening procedure (items 1-5, see below). The same questionnaire is sent to all subjects not responding to the first invitation (non-respondents), together with the reminder, and to all non-participants to obtain information about the reasons of non-participation. In case of low response, 10% of non-respondents will be contacted by telephone to assess a shortened version of this questionnaire. A second questionnaire is sent to participants two weeks after the screening test and will contain questions about the perceived burden of the screening method (item 5).

#### 1) Demographic and socioeconomic status

Baseline characteristics as age, gender, marital status, ethnicity, education and employment will be collected.

#### 2) Satisfaction of the prior consultation

To evaluate the satisfaction of the prior consultation by telephone compared with the prior consultation at the outpatient clinic, items are included based on an existing patient-satisfaction questionnaire[45].

#### 3) Reasons for (non-)participation and understanding of the information leaflet

In the first and second Dutch pilots on screening for colorectal cancer with FOBT,[36,46,47] a questionnaire has been developed to collect data on awareness of CRC, reasons for (non-) participation and clarity and readability of the information leaflet. As in the second round of FOBT screening, the Health Belief Model (HBM) is used as theoretical background to understand the reasons for (non-) participation[36,48]. The corresponding items are adjusted for this study.

#### 4) Informed choice

To evaluate the proportions of participants and non-participants who made their decision on the basis of an informed choice, it is required to assess the knowledge, attitude and uptake of the invited persons. Items concerning knowledge and attitude are derived from both Dutch FOBT pilots [13,36], and from the evaluation of prenatal and lung cancer screening in the Netherlands which is based on Marteau's measure of informed choice [49-51].

#### 5) Expected and perceived burden of the screening procedure

The experience of participants with the screening procedure is evaluated with a questionnaire assessing anxiety, embarrassment, pain and discomfort. The items assess the perception of participants regarding the bowel preparation and the burden of the screening procedure itself. Responses are scored on a standard formatted five-point Likert scale. Satisfaction with the screening procedure will be measured by items scored on a 4-point scale.

This questionnaire is based on questionnaires used in the first and second Dutch pilot for screening with FOBT[34,36] and on studies investigating the acceptance of CT colonography[34] and patient perception of diagnostic tests for faecal incontinence[52], as well as on previous discrete choice experiments showing that especially type of bowel preparation, risk education of CRC related death and length of screening interval influence CRC screening preferences[37].

### Cost-effectiveness

Cost-effectiveness will be evaluated by incorporating the final results in the validated MISCAN-colon screening model for cost-effectiveness. That model compares different methods of CRC screening in our country and is used to estimate the costs, colonoscopy and CT colonography capacity requirements and effects of colonoscopy and CT colonography screening versus other screening programmes[53].

### Ethical approval

Ethical approval was obtained from the Dutch Health Council (2009/03WBO, The Hague, The Netherlands).

### Data analysis

We will calculate the participation rate as the number of participants undergoing the screening test relative to the total number of all eligible invitees. We will compare the participation rate between the three screening programmes, and express it as relative participation rates, calculating corresponding 95% confidence intervals, using colonoscopy screening with a prior consultation at the outpatient clinic as the reference strategy.

We will test the null hypothesis of no difference in participation rate using chi-square test statistics. We will also calculate a conditional relative participation rate using logistic regression modelling, accounting for baseline variables. If the overall hypothesis of no difference is rejected, we will test the null hypothesis of a difference in mode of invitation to direct colonoscopy. Additionally, we will test the null hypothesis of no difference in participation between CT colonography screening and direct colonoscopy screening.

The detection rate of the screening test is defined as the proportion of screenees with detected advanced neoplasia. Advanced neoplasia comprises all carcinomas and advanced adenomas together. An advanced adenoma is defined as an adenoma ≥ 10 mm, with villous histology (≥25% villous) or with high grade dysplasia. The most advanced detected lesion per screenee will be used to calculate the detection rate.

The diagnostic yield per 100 invitees is defined as the proportion of screenees with detected advanced neoplasia relative to all eligible invitees.

### Sample size

We anticipate a participation rate of 22.5% in colonoscopy screening with a prior consultation at the screening centre, a participation rate of 27.5% with prior consultation by telephone, and a participation rate of 35% in the CT colonography group (based on the participation rate of FS screening in the Netherlands)[17]. Including 7,500 participants in the trial will then achieve a power exceeding 99% to reject the null hypothesis of no difference, using a 2 degrees of freedom Chi-Square test with a significance level set at 0.05.

These numbers will lead to a 98% power to reject subsequently the hypothesis of no difference in participation for the two consultation modes with direct colonoscopy, and a power exceeding 99% for the two other pair wise comparisons between screening programmes.

## Discussion

The optimal screening test is still under debate. Stool-based tests and FS are considered as suboptimal tests. DCBE and capsule endoscopy are not recommended for CRC screening, because of its inferior diagnostic sensitivity.

Colonoscopy and CT colonography are the most accurate colonic exams and screening options by which inspection of the entire colon is allowed. Prospective data on the effectiveness (CRC mortality reduction) of population based screening programmes by colonoscopy and CT colonography are lacking. The effectiveness of a screening programme is directly influenced by the participation rate. It is expected that the participation rate of CT colonography screening is higher than with colonoscopy screening, because of the lower burden of the procedure. However, all CT colonography positives will need colonoscopy for confirmation and therapy or need surveillance which can lower the participation rate to the total CT colonography programme. It is not known to what extent this would influence participation rate.

This study will evaluate the efficacy of a colorectal cancer screening programme in the Netherlands using colonoscopy and CT colonography as a screening method. It will show whether participants are well-informed about the screening programme and what factors would influence (non-)participation. If this study shows that a prior consultation by telephone instead of a prior consultation at the outpatient clinic in colonoscopy screening has a positive effect on the participation rate, this would contribute to the effectiveness of future screening programmes.

This study will provide information on the uptake of a population screening by colonoscopy and CT colonography and on factors influencing the uptake. Furthermore, this study will give insight into concerns on CT colonography and especially on colonoscopy screening including burden of the procedures, risks and costs. As other screening options have already been investigated, this study will enable to determine the most cost-effective screening method for CRC in the Netherlands.

## Abbreviations

CRC: colorectal cancer; AMC: Academic Medical Centre; Erasmus MC: Erasmus MC University Medical Centre; FOBT: faecal occult blood test; gFOBT: guaiac-FOBT; iFOBT: immunochemical-FOBT; sDNA: DNA stool tests; FS: flexible sigmoidoscopy; CT colonography: computed tomography colonography; DCBE: double contrast barium enema; IBD: inflammatory bowel disease; HBM: health belief model.

## Competing interests

The authors declare that they have no competing interests.

## Authors' contributions

TRW, MCH and EMS are responsible for the drafting of the manuscript. MD, PF, PMMB, MT, MB, MLEB, MEL, EJK, ED and JS are responsible for the study design and revision of the manuscript. All authors have read and approved the manuscript.

## Pre-publication history

The pre-publication history for this paper can be accessed here:

http://www.biomedcentral.com/1471-230X/10/47/prepub
